# Cooperation Achieves Results at UNC-CH

**DOI:** 10.1289/ehp.112-1277129

**Published:** 2004-08

**Authors:** Mary Eubanks

Now that scientists have solved the code for the human genome and are moving forward to sequence the genomes of other organisms, a significant challenge lies in how to harness this vast amount of new information to benefit society. Recognizing that cross-communication and cooperation among different research labs is essential to realizing the potential of genomic science for toxicology, the NIEHS earmarked $37 million to establish the Toxicogenomics Research Consortium (TRC) in November 2001.

A major objective of the TRC is to link investigator-initiated research projects at five academic centers—the Massachusetts Institute of Technology, Duke University, the University of North Carolina at Chapel Hill (UNC-CH), Oregon Health & Science University, and the Fred Hutchinson Cancer Research Center/University of Washington—in a collaborative effort to understand how organisms respond to chemical exposures and other kinds of environmental stress. At UNC-CH, investigator-initiated research projects focus on different model systems—from mice and cell lines to humans—all with a common, unifying focus on environmental challenges that cause DNA damage or oxidative stress, leading to cancer.

With the goal of finding molecular signatures that can be used for early risk assessment, these projects employ microarray tools to assay for gene expression responses to cancer-related chemicals. Some use the same compounds that cancer patients are already receiving, to gather information on how different populations may react to certain drugs.

## Dampening the Background Noise

In one TRC projects, David W. Threadgill, an assistant professor of environmental sciences and engineering, uses mouse models to investigate differences in response to various tumor-inducing and chemotherapeutic chemicals from one mouse strain to another. In his model, all the mice of one strain are essentially identical, providing an unlimited number of individuals that have the same genotype. This experimental approach enriches the opportunity to separate out what is “noise” in the data from what is truly meaningful. Threadgill’s lab is currently profiling about a dozen mouse strains, some of which are susceptible to certain types of cancer while others are resistant.

Among organisms, there is a wide range of susceptibility to the effects of different toxicants. The classes of chemical compounds that cause cancer—including alkylating agents, DNA-damaging agents, and toxicants that affect the cell cycle—vary in the mechanisms by which they cause the disease. There is also extensive variation in the way different cancer patients respond to treatment and how they handle the toxic effects of different chemical therapies.

To better understand why this is so, Threadgill exposes different mouse strains to various chemicals over a variety of dose regimens and time spans, then compares the results to a control group. After exposure, tissue is harvested from the liver, colon, and breast for extraction of RNA that is converted into cDNA. The cDNA is hybridized to microarray chips containing 17,000 mouse genes. If a gene is turned on or up as a result of exposure, it will fluoresce more brightly than the same gene on the corresponding chip for the control group. Analysis of the signals reveals which genes increase their expression level as a result of exposure to a particular toxicant and which genes are turned down.

Threadgill explains, “Lots of genes are turned on, up, or down. Comparison of the signals from different strains reveals which genes are unique to certain classes of compounds, at what dose levels and time treatments.” His lab also examines direct causes of the associated physical changes and their correlation with prior knowledge of how certain classes of compounds affect tissue. He has detected significant strain-dependent differences in gene expression in both baseline and treated colon tissue. He says, “The characterization of these differences in the context of disease and treatment end points should reveal new insights into the mechanistic causes of the cancer end points.”

Screening thousands of genes and expression changes presents a huge computational challenge to discern what the critical signatures are. Biostatisticians have begun to mine the data collected by Threadgill over the first two and a half years of his project to evaluate thousands of signals and identify similarities and differences between treatment time courses, exposure levels, and individual strains. Knowing which genes are significant is vital to finding prognostic markers for whether an individual is susceptible to effects from certain compounds and for evaluating what makes certain individuals more susceptible than others.

## Layers of Response

In a coordinated experimental design, Charles M. Perou, an assistant professor of genetics, is screening some of the same compounds as Threadgill in his investigations of the transcriptional response in breast cancer using human cell lines from breast tumors and immortalized breast epithelial cell lines (that is, publicly available cell lines that have been maintained over time for experimental use). This may allow for comparative analyses of results across different model systems that could lead to new insights into the resulting biological insults from exposure to specific toxicants. Cell lines are treated with different regimens, and then examined for gene expression changes using microarrays.

Perou is also studying the effects of these drugs on real breast tumors of patients before, during, and after chemotherapy for comparison of similarities or differences in experimental cell lines with tumors in patients. In a paper in the June 2004 issue of *Cancer Research,* Perou’s lab reported that a small but significant set of genes were identified as being changed in both *in vitro* cells and living tumors, and that these are likely to be very important in cellular response to toxicants. In this study, 20–30 changes observed in the expression profiles identified potential candidate genes for a general response to these compounds.

Perou and colleagues have discovered that although the compounds tested have different mechanisms and targets in cells, the overall cellular response to the different drugs is the same—a sort of dominant generic stress response that is similar across genes. The secondary drug-specific response pattern is layered on top of this generic stress response pattern. Perou says, “Arrays do a great job of implicating genes in certain processes in response to different drugs, but the genes involved in the response are the same. . . . This leads to the hypothesis that some people with reduced ability to respond might be more susceptible because of variants in these generic stress response genes.”

Perou’s work has also uncovered unique molecular signatures that distinguish two subtypes of breast cancer. Patients with one type will do well under the standard drug therapies and survive. However, for those with the other type, prognosis for survival if given standard treatment is not good; more aggressive treatment such as radical mastectomy and radiation would be needed, and even then the chances of survival are not nearly as good. This complex overlapping pattern of drug-specific response genes embedded within the common response will be reported in a second paper submitted for publication.

Perou is particularly interested in the generic stress phenomenon because individuals who have susceptible variants of these genes could be unable to respond to many different toxicants in their environment. He says, “Microarrays provide a big list of candidate genes. The challenge is to figure out [which genes] are the passengers and [which] are the drivers, then apply that information to population studies to identify genetic variants that are hyperactive and correspond to susceptibility.” Through the TRC, he says, it may ultimately be possible to link findings and design experiments such that scientists can identify a common set of genes in mice and humans for independent validation for susceptibility. “As we learn more about evolutionarily conserved genes across species that are involved in reactions to environmental chemicals,” Perou says, “we will have more confidence in the scientific findings.”

## Confidence Building

William K. Kaufmann, director of the UNC-CH Program in Toxicogenomics and director of the Genetic Susceptibility Research Core in the UNC Center for Environmental Health and Susceptibility, studies expression of tumor suppressor genes in the cell cycle. Kaufmann is interested in how changes in the DNA at the ends of chromosomes provide life span checkpoint monitors that signal the time for aging cells to die. Experimental evidence indicates that responses similar to those involved in cell senescence occur in breast, liver, and colon cells exposed to carcinogenic compounds. Such changes are accompanied by reproducible gene expression patterns.

Kaufmann says, “When the same unexpected patterns of response are seen in lines from several different individuals, our confidence that the response to stress is biologically meaningful increases.” Knowledge gained from the characterization of modulations in expression of genes as a result of exposure to different carcinogens could one day provide a fingerprint to enable design of precise treatment strategies for specific individuals exposed to different toxicants.

As the robustness of microarray technology for gene expression profiling improves and becomes more standardized, scientists will be able to employ molecular signatures to tailor therapies as well as conduct exposure risk assessment. Kaufmann concludes that in spite of sometimes feeling overwhelmed by too many genes and the need for a bigger computer, the UNC-CH microarray research projects are producing a data-rich picture, and are spawning whole new areas of discipline to look for patterns of change. Such individual scientific discoveries and applications of the principles of toxicogenomics are resulting in a serendipitous research crossover that will continue to spur insights, leading to faster, broader, and more effective applications for public health.

## Figures and Tables

**Figure:**
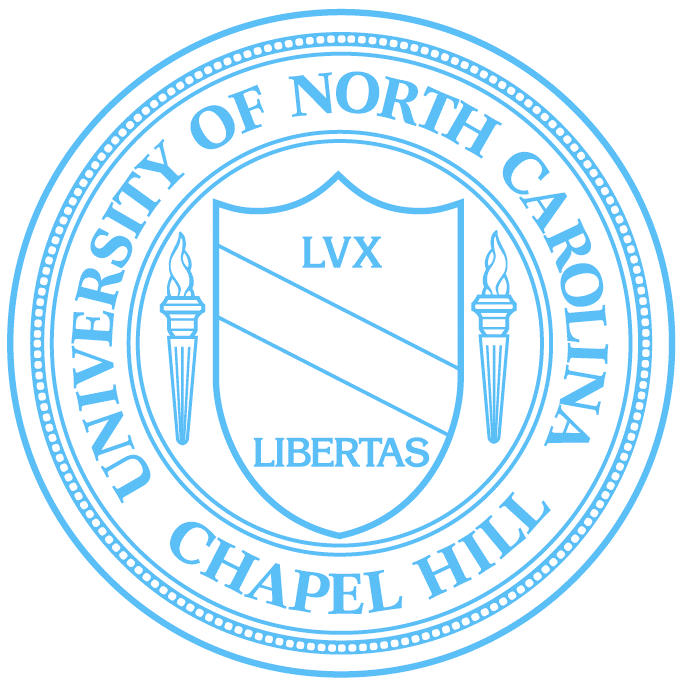


**Figure:**
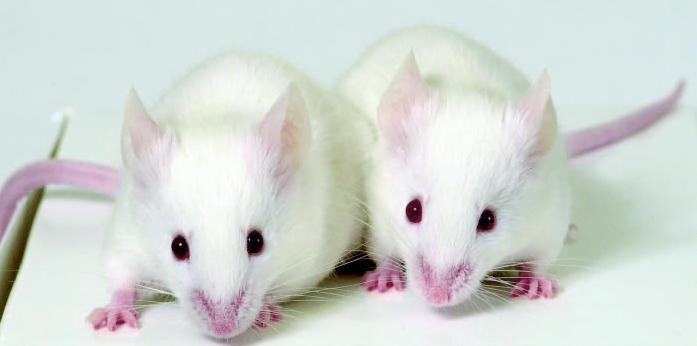
**A perfect likeness.** UNC-CH researcher David W. Threadgill uses strains of mice in which all the animals have the identical genotype to study cancer susceptibility from exposure to a variety of environmental toxicants. Some of the mouse strains are resistant to cancer, while others are more susceptible to the disease.

**Figure:**
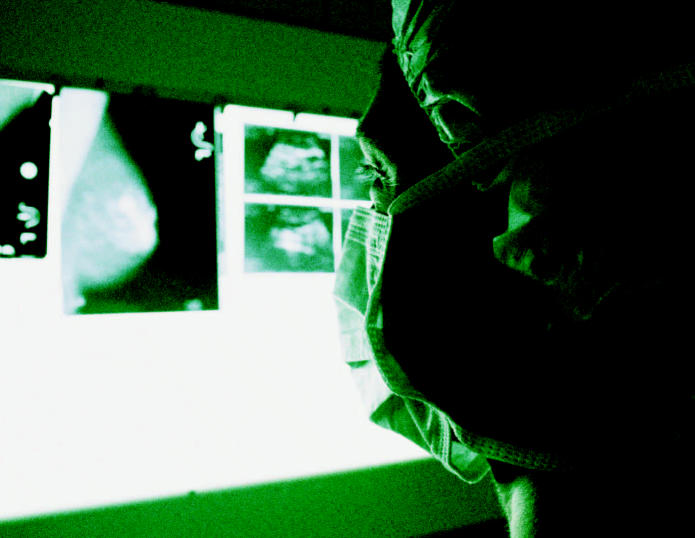
**Molecular mysteries.** UNC-CH researchers are elucidating how susceptibility to breast cancer is impacted by a “generic” stress response that is similar across genes. Such a generic response underlies characteristic drug-related responses, which molecular signatures indicate vary from person to person.

